# ADONHERS (Aged DONor HEart Rescue by Stress Echo) National Protocol: Recipient’s Survival after 10-Year Follow-Up

**DOI:** 10.3390/jcm12103505

**Published:** 2023-05-16

**Authors:** Giulia Elena Mandoli, Maria Barilli, Davide Soviero, Nicolò Ghionzoli, Federico Landra, Massimo Maccherini, Sonia Bernazzali, Benedetta Maria Natali, Marta Focardi, Luna Cavigli, Flavio D’Ascenzi, Maria Concetta Pastore, Carlotta Sciaccaluga, Tonino Bombardini, Serafina Valente, Matteo Cameli

**Affiliations:** 1 Department of Medical Biotechnologies, Division of Cardiology, University of Siena, Policlinico “Le Scotte”, 53100 Siena, Italy; 2 Cardio-Thoracic and Vascular Department, Cardiac Surgery Unit, University of Siena, Policlinico “Le Scotte”, 53100 Siena, Italy; 3 Institute of Clinical Physiology, National Research Council, 56124 Pisa, Italy; bombardini@ifc.cnr.it

**Keywords:** advanced heart failure, heart transplant, marginal heart donors, stress-echocardiography

## Abstract

**Background**: The gold-standard treatment for end-stage heart failure is heart transplantation, but the lack of organ donors remains an important limitation in this field. An accurate selection of marginal hearts is fundamental to increase organ availability. **Purpose**: In our study we analyzed if recipients receiving marginal donor (MD) hearts, selected by dipyridamole stress echocardiography according to the ADOHERS national protocol, had different outcomes compared to recipients with acceptable donor (AD) hearts. **Methods**: Data were collected and retrospectively analyzed from patients who received an orthotopic heart transplant at our institution between 2006 and 2014. Dipyridamole stress echo was performed on identified marginal donors and selected hearts were eventually transplanted. Clinical, laboratory and instrumental features of the recipients were evaluated and patients with homogenous baseline characteristics were selected. **Results**: Eleven recipients transplanted with a selected marginal heart and eleven recipients transplanted with an acceptable heart were included. Mean donor age was 41 ± 23. The median follow-up was 113 months (IQR 86–146 months). Age, cardiovascular risk and morpho-functional characteristics of the left ventricle were comparable between the two populations (*p* > 0.05). Left atrial size was significantly higher in patients with marginal hearts (acceptable atrial volume: 23 ± 5 mL; marginal atrial volume: 38 ± 5 mL; *p* = 0.003). Acceptable donor recipients showed a higher impact of Cardiac Allograph Vasculopathy (*p* = 0.019). No rejection differences were found between the two groups. Four patients deceased, three were standard donor recipients and one was from the marginal donor group. **Conclusions**: Our study shows how cardiac transplant (Htx) from selected marginal donor hearts through a non-invasive bedside technique can alleviate the shortage of organs without a difference in survival compared to acceptable donor hearts.

## 1. Introduction

In the field of heart failure (HF), 1 to 10% of European patients’ progress to the stage of advanced HF was characterized by signs and symptoms that are not responsive to medical therapies [1,2]. The overall number of patients with advanced HF has been growing due to the amelioration of therapies and the ageing of the population, but 1-year survival prognosis still ranges from 25 to 75% [3]. Mechanical circulatory support (MCS) can represent a valid option when these patients reach the stages 1 to 4 as classified by the Interagency Registry for Mechanically Assisted Circulatory Support (INTERMACS), but the survival rate after 2 years from the implant remains low [4,5]. In fact, even though the last few decades have shown fast improvement in the field of long-lasting mechanical circulatory support (MCS), the gold-standard therapy for these patients remains a heart transplant (Htx), with a median survival of 12.5 years and a markedly increased quality of life [3,6,7]. Lack of organ donors remains one of the main limitations for patients awaiting HTx, while the number of patients with end-stage heart failure on the waitlist keeps growing worldwide.

From the definition by Copeland et al. in 1995 [8], heart donor criteria have been extended in the attempt to reduce the Htx waitlist. The use of marginal donors (MD), defined as a donor with organs that did not comply with the standard criteria, became a necessity when it became clear that the risk of death on the waiting list for end-stage HF patients was higher than the benefit of receiving a MD heart. This limitation led clinicians toward the finding of appropriate techniques to perform a comprehensive evaluation of cardiac organs without optimal characteristics and enlarging the availability of donor hearts. Marginal organs have been described as hearts collected from donors with an elevated burden of risk factors such as advanced age, discrepancy of size with the receiver, prolonged ischemic time, the presence of left ventricular hypertrophy or mild dysfunction, long use of inotropes and infection with hepatitis B/C (Table 1) [9,10]. No standard definition of MD has yet been developed, posing an important bias for accurate research. So far, the main works conducted on MD have shown how a detailed assessment of donor left ventricular (LV) dysfunction allowed an increase of utilization rates from 38–59% in recent years [11,12]. Worldwide, the main reasons for exclusion from the donor list are age of donor over 55, prolonged ischemic time over 4 h and mild LV dysfunction [13]. Thus, the only existing ongoing prospective and multi-center trial investigating marginal hearts includes donors with expected total ischemic time ≥ 4 h or expected total ischemic time ≥ 2 h and at least one of the following: left ventricular hypertrophy, EF 40–50%, downtime ≥ 20 min, donor age > 55 years. An accurate and systematic selection of organ function is therefore essential in this field and many techniques have been evaluated. One of the available tools that has been used and described to assess sub-optimal hearts is stress echocardiography [14]. Global or regional left ventricular dysfunction are common after brain death, but the damage has been proven to be reversible without a negative effect on recipients’ prognosis [15]. The recent International Society for Heart and Lung Transplantation (ISHLT) guidelines introduce pharmacological stress echocardiography to assess dysfunctional MD with a Class of recommendation IIa, Level of evidence C [16]. The technique has been shown to be useful in three different settings: (1) donors aged > 55 and normal LV function; (2) donors with mild LV dysfunction that improves in the first minutes of echo-stress; (3) donors with global or regional LV dysfunction that slowly improves (hours) with hormonal stimulus [17,18,19,20,21,22,23,24]. Promising results and favorable outcomes for the recipients have been described after the selection of hearts through non-standard/second level echocardiography techniques such as stress echocardiography, without negative effects on the medium- and long-term success [25]. Moreover, in hearts that are not eligible for transplant after a stress test, the presence of coronary artery disease was revealed through cardioautoptic confirmation [22].

**Figure 1:**
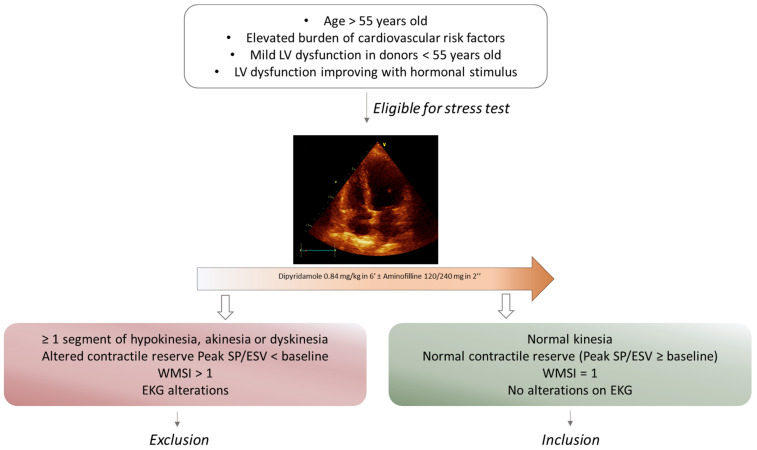
Flow-chart for the selection of marginal heart donors after bedside echocardiography. Patients aged > 55 years or aged < 55 with cardiovascular risk factors or mild LV dysfunction were chosen to undergo dipyridamole stress echocardiography. Criteria of exclusion or inclusion of detected hearts eligible or not eligible for heart transplantation. *ESV: end-systolic volume; LV: left ventricle; SP: systolic pressure; WMSI: wall motion score index*.

The “ADONHERS” protocol was created to decrease shortage of donor hearts by starting a national network for the research and evaluation of hearts collected from donors over the age of 55 or under the age of 55 with cardiovascular risk factors (diabetes, arterial hypertension, pre-mature coronary artery disease, cocaine or amphetamine abuse, drug intoxication, smoking history). This network includes the involvement of cardiosurgical transplantation centers, interregional levels (AIRT, NITp and OCST) and neuroresuscitation centers, that statistically provide the highest number of donations. After declaration of brain death and notification from the center of donation, patients were screened through clinical evaluation and analysis of clinical history. LV function was then analyzed by an expert cardiologist with standard bedside echocardiography. After meeting inclusion criteria (Figure 1), hearts were evaluated through dipyridamole stress echocardiography and eventually approved for transplantation.

The aim of this study was to analyze recipients that received marginal donor hearts, selected by dipyridamole stress echocardiography and collected from a network of intensive care units (ICU) all over the region, and compare outcomes with recipients that received acceptable donor hearts.

## 2. Methods

### 2.1. Patient Selection and Data Collection

In the present retrospective study, we collected the data of patients who received an orthotopic transplant between 2006 and 2014: eleven were transplanted with a selected marginal heart and the remaining were transplanted from hearts whose characteristics fit into the selection of optimal donors. Clinical, laboratory and baseline echocardiographic features of the two groups of recipients were evaluated and patients with homogeneous baseline characteristics were selected to reduce biases among the two groups (Table 2). Once informed consent was obtained, data were collected from the ambulatory of advanced HF and heart transplant of the University Hospital “Le Scotte” of Siena, where patients performed routine surveillance with serial endomyocardial biopsies and coronary angiography according to guidelines [26]. All echocardiographic examinations were performed by experienced operators using GE Vivid iq Cardiovascular ultrasound following to the recommendations of American Society of Echocardiography/European Association of Cardiovascular Imaging [27,28]. The study was performed in accordance with the Declaration of Helsinki.

### 2.2. Marginal Hearts Selection

The selection of marginal hearts was realized following the ADONHERS protocol [29] (Figure 1) from 2006 to 2014 when candidate donors were enrolled. After legal declaration of brain death, standard bedside echocardiography was performed to select hearts suitable for stress echocardiography. The exclusion criteria were the presence of sub-optimal basal echocardiographic acoustic window, regional wall motion abnormalities or global ventricular dysfunction (LV ejection fraction [EF] < 45%), diastolic dysfunction grade II or greater, ventricular parietal hypertrophy (interventricular septum > 13 mm, left ventricular mass index > 175 g/m^2^), and moderate to severe valvular disease. None of the donors underwent inotropic therapy before heart explant during hospitalization in intensive care units. Dipyridamole stress echo was than performed on suitable hearts following the European Association of Echocardiography (EAE) protocol with the scheme 0.84 mg/kg in 6 min [30]. When the test resulted normal, marginal recipients were uneventfully transplanted and coronary angiography at follow-up confirmed no coronary lesions.

### 2.3. Statistical Analysis

Statistical analysis was performed with software IBM SPSS 25.0 (1989-2017, LEAD Technologies Inc., Charlotte, NC, USA). Distribution of continuous variables was detected using the Kolmogorov–Smirnov normality test. Continuous variables with normal distribution are represented as mean deviation.

Standards and their significance were analyzed by the Student T test for independent samples. Non-standard continuous variables were represented as median (interquartile range) and their significance was analyzed by the Mann–Whitney test. Non-continuous variables were represented as frequency (percentage), and the relative significance was tested by the Chi-framework test or the Fischer test, as appropriate. In all cases data were considered significant with values of *p* < 0.05.

## 3. Results

Twenty-two recipients were included in the study, eleven with a marginal donor heart (group 1) and eleven with an acceptable donor heart (group 2). The median follow-up was 113 months for both groups. Among donors, the median age was 41 ± 23 years, being lower in group 1 than in group 2 (45 ± 29 years vs 36 ± 12 years). Ten patients were females (47.6%) of whom six were marginal donors (54.5%).

The cohort of recipients had a median age at transplantation of 54 ±10 years, without a significant difference between group 1 (55 ± 10 years) and the control group (54 ± 9 years). Eight recipients were female (36.4%), equally divided into the two groups. The average body mass index (BMI) was 29 ± 5.9 Kg/m^2^ with a 30.1 ± 7.2 Kg/m^2^ BMI in group 2. Class NYHA III/IV was retrieved post-transplant only in three patients belonging to the cohort of recipients of acceptable hearts, while no dysfunctional class was recorded among patients who received marginal hearts. Concerning the stratification of cardiovascular risk between the two groups, 10 patients had arterial hypertension (58.8%), of which 6 were part of the control group (60%). Four were affected by diabetes mellitus (18.2%), of which three were from the control group (27.3%) and one was from group 1 (9.1%). A total of 35.3% of patients suffered from dyslipidemia, with a higher prevalence in the control group (55.6%) compared to 9.1% of marginal heart patients. Chronic kidney disease affected 11 patients, of which 7 were in group 2 and 4 in group 1 (Table 2).

**Figure 2:**
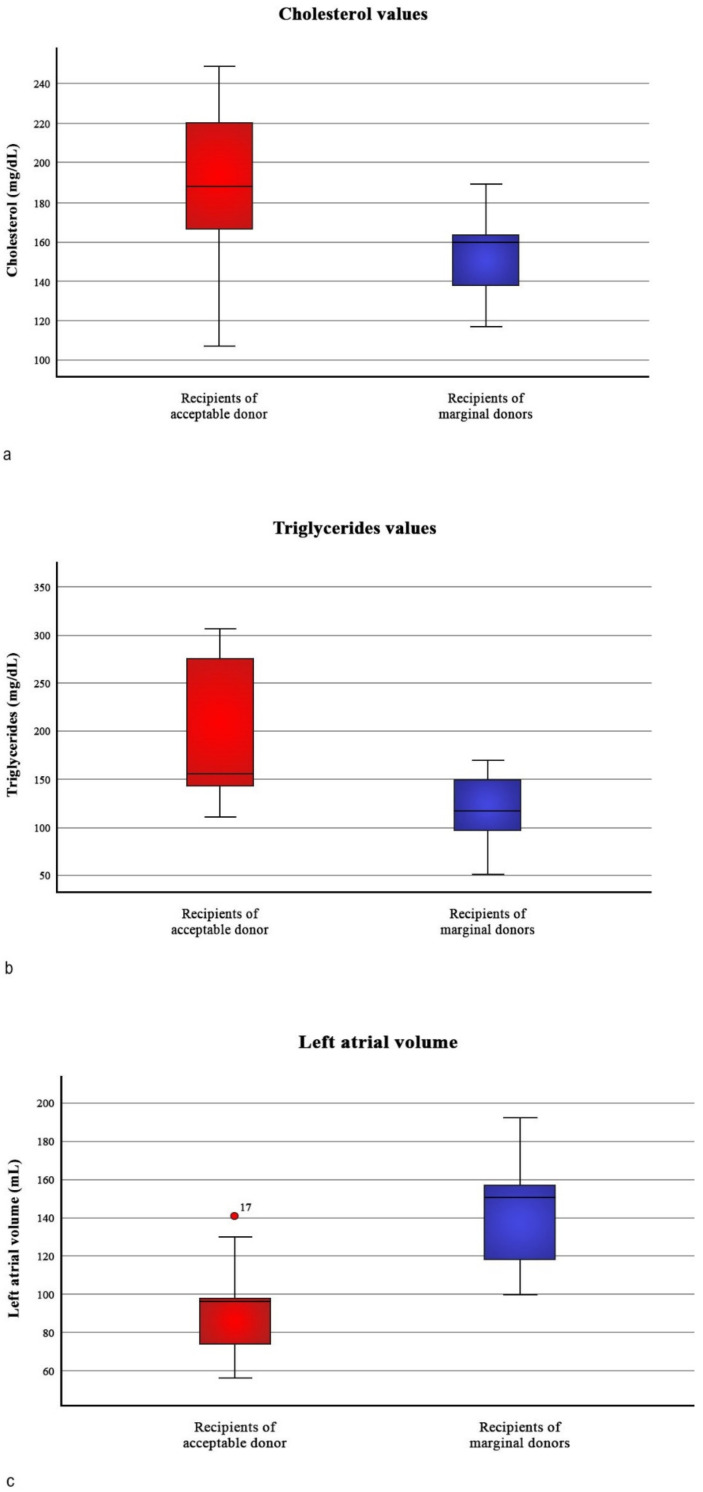
Differences in (**a**) cholesterol, (**b**) triglycerides, (**c**) left atrial volume during follow-up among recipients of acceptable hearts and recipients of marginal hearts.

At follow-up visit, blood tests were carried out regularly and analyzed. Differences in white blood cells, NT-proBNP, ultra-sensitive troponin and creatinine were not statistically relevant between the two groups. Conversely, the levels of total cholesterol and triglycerides were significantly higher in the control group with values of 189 ± 44.7 mg/dL vs. 155 ± 22.3 mg/dl (*p* = 0.048) and 196 ±77 mg/dL vs. 118 ± 37 mg/dl (*p* = 0.012), respectively (Figure 2a,b).

Echocardiography was performed following the routine protocol of our center. The two groups turned out to have homogeneous features for size and function of the left ventricle, either systolic or diastolic (*p* > 0.05). Left atrial size was significantly higher in marginal hearts (Optimal heart atrial volume: 23 ± 5 mL; Marginal heart atrial volume: 38 ± 5 mL; *p* = 0.003) (Figure 2c).

**Figure 3:**
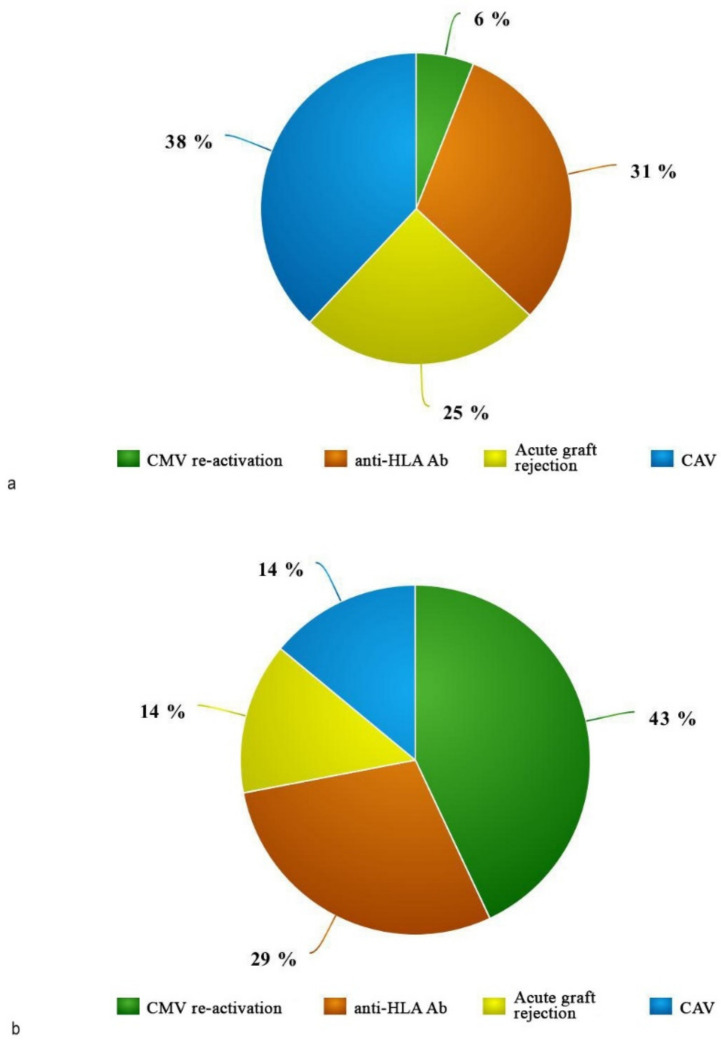

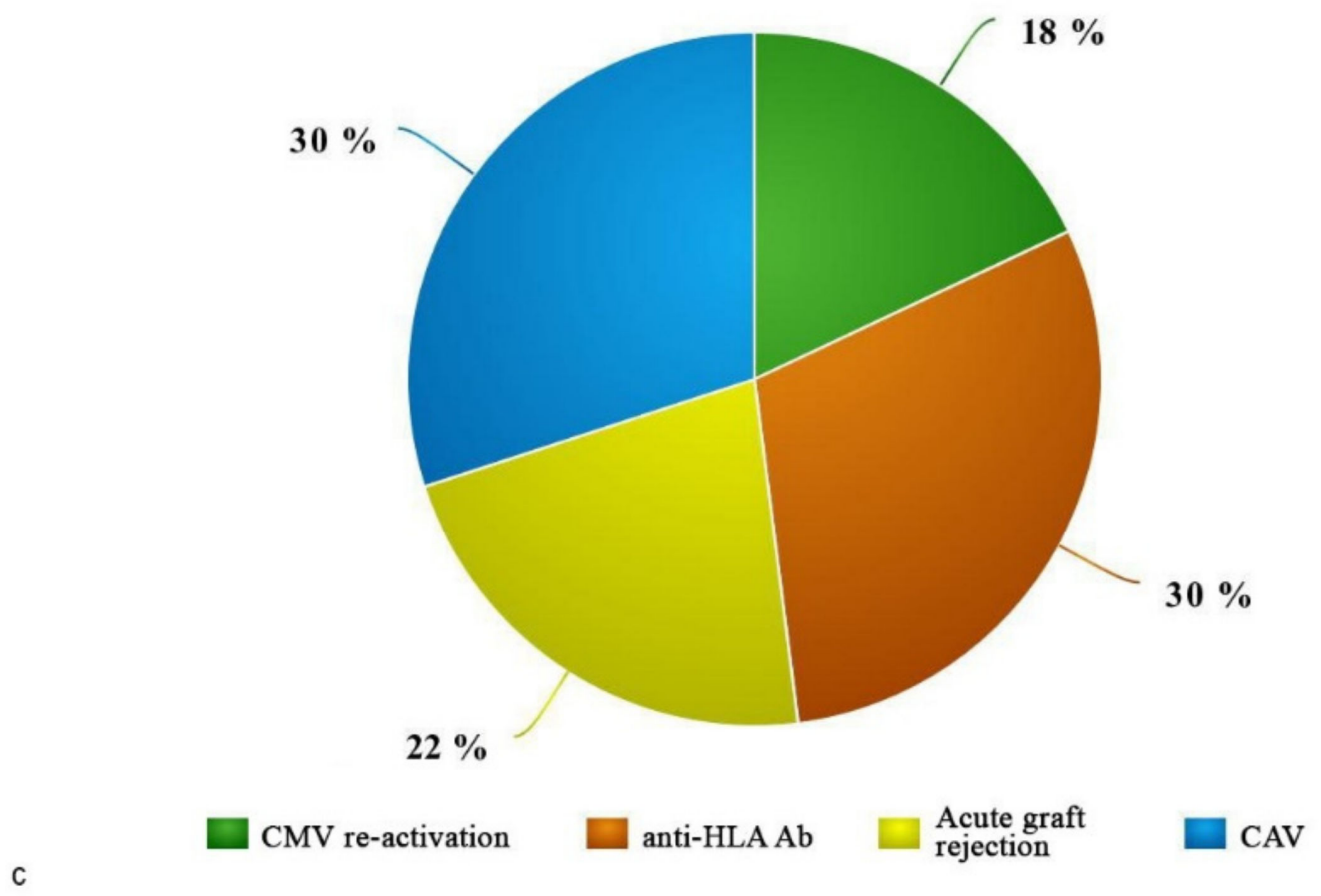
Major post-transplant complications retrieved in the two populations analyzed. (**a**) shows the distribution of Htx complications in acceptable donor recepients, (**b**) shows the same in marginal donor recipients and (**c**) shows the percentage of Htx complications in the overall population.

Immunosuppressive therapy was set following ISHLT protocol [26,31]. During the follow-up, the regimens were changed in relation to the development of adverse effects (e.g., kidney injury, Coronary Allograft Vasculopathy, rejection, etc). Glucocorticoids and tacrolimus were mainly used in the group transplanted with optimal hearts while mycophenolate mofetil and cyclosporine were mainly used in the marginal recipients group. After HTx, treatment with lipid-lowering drugs was set only in 72.7% of patients. Regarding the most common complications after HTx (Figure 3a–c), a degree of rejection was recorded in 5 patients, of which 4 belonged to the control group. Moreover, 7 patients developed antibodies directed against the donor’s HLA, most of them in the control group. Cardiac allograft vasculopathy (CAV) was the most frequent complication. A total of 31.8% of patients developed CAV with a significantly higher incidence in the control group than in the marginal group (*p* = 0.019).

## 4. Discussion

Since the first successful heart transplant in 1967, the original traditional criteria [18] used to identify appropriate heart transplant donors has changed due to the undersupply of available organs [10,13]. Nonetheless, the waiting list time and the demand for cardiac organs has kept growing in the last 20 years [32,33]. Looking at the 2020 Annual report by the Scientific Registry of Transplant recipients, from 2009 to 2020 there was an increase of 32.5% in the number of enrolled listings with older candidates waiting in the lists. Moreover, with ventricular-assisted devices (VAD) taking hold in this scenario, the proportion of patients waiting with a VAD grew from 16% to 36% [33]. Italian data collected from the period 2000–2018 show a median waiting time for patients on the list of 38 months with a percentage of transplants in 2018 of 32.5%. During the same year, of the 717 people on the waiting, list 53 died, with a yearly death rate of 7.9%. Of all the hearts transplanted in this time frame, only 5–10% of donors were aged over 55 [32]. The supply of donor hearts is strongly reduced by this cut-off limit assigned at 55 for the prevention of coronary artery disease (CAD) and worse results after transplant [34,35,36,37]. However, there is evidence of the equivalent survival of recipients transplanted with the hearts of a donor older than 50 [38,39,40], especially when considering the benefit of transplant against the risk of remaining on the waiting list [39,40].

A 6.5-year analysis from the United Network for Organ Sharing (UNOS) database displayed how almost 20% of potential donors each year are excluded due to a dysfunctional left ventricle after brain death. With a longer evaluation, Huckaby et al. showed that of all the transplants realized from 2009 to 2019, only 20.6% were from MD, but no clear definition for extended criteria permitted a valuable analysis of their impact on Htx lists. The highest proportion of marginal organs were collected from donors with an ischemic time longer than 240 min, without a different trend in the usage of over-55 donors or with borderline ventricular function [13]. With a great number of donors excluded from the pool due to their advanced age and around 20% of cardiac organs excluded due to LV dysfunction after brain death, the recruitment of even a small part of this cohort of hearts could greatly increase shortage of organs [18,41,42]. The ISHLT consensus statement on heart procurement suggest performing coronary angiography in potential heart donors with risk factors of CAD (including age) [43], but its application is not always feasible and can be a cause of delay. Thus, the latest 2022 ISHLT guidelines for donor heart selection implement other imaging techniques for a correct and precise assessment of MDs with higher risk of CAD.

The ADONHERS protocol has been created to extend the possibility of donation to older potential donors by performing a non-invasive and inexpensive technique such as pharmacological stress echocardiography. It can be applied when a marginal donor is identified by a Transplant Coordination Center and selected by precise clinical and bedside echocardiography criteria. The first results of the project showed remarkable outcomes with a 1-year survival post-marginal heart transplant of 93% [17,44]. Stress echocardiography is proven to be an efficient tool to assess systolic and diastolic function, either in case of subclinical CAD, cardiomyopathy or stunned myocardium under hormonal treatment [22,45]. It allows the evaluation of inducible ischemia by the examination of segmental wall motion with the regional wall motion score index (WMSI) and alterations on ECG. The contractile reserve is analyzed through LV elastance using the formula of systolic pressure/end-systolic volume index, with a defined cut-off of enhancement of 5% [29]. The EAE protocol for stress echo in this setting involves the use of dipyridamole, because of its steal phenomena through arteriolar vasodilation that does not involve catecholamines. Dobutamine is indicated only in cases of strong contraindications for dipyridamole because its effect could be detrimental by inducing direct myocardial injury and/or coronary vasospasm [22,44].

Our work is a single-center pilot study that compared outcomes and survival in patients transplanted with echo stress-selected marginal hearts or with acceptable hearts. In particular, our analysis showed the absence of statistically significant difference in terms of mortality between the two examined groups (*p* = 0.269). The two cohorts of recipients showed homogeneous characteristics when looking at cardiovascular risk factors. At follow-up, laboratory and echocardiographic data were found to be comparable with equal therapeutical protocol. Interestingly, the only differences recorded at basal echocardiography concerned left atrial dimensions, even if the diastolic functional parameters were overlapping. The reasons for this result could be several, including differences in surgical technique and physiological divergencies in atrial dimensions of marginal and acceptable donors. Marginal donors are usually affected from higher cardiovascular risk factors, especially arterial hypertension, a known cause of atrial enlargement. This hypothesis needs to be validated with further analysis, including morpho-functional features of donor hearts before collection.

Despite the fact that our study did not show considerable differences in survival, unexpectedly, CAV incidence was found to be significantly higher among optimal donor recipients in comparison to marginal donor recipients. In our cohort of study, this finding did not affect mortality, even though the negative prognostic impact of CAV in HTx is widely known [46,47,48]. Furthermore, the presence of CAV might affect patients’ quality of life in relation to the development of symptoms, especially dyspnoea or impaired functional capacity, when systolic and/or diastolic function are affected. An additional finding was the relation between incidence of CAV and higher values of total cholesterol and triglycerides in recipients of optimal hearts. This result strongly underscores the need to implement an hypolipidemic drug regimen also in heart-transplanted patients. In particular, statins should be used not only to reduce lipid levels in the blood but also because evidence suggests possible immunomodulatory effects carried out by the use of statins [49].

Finally, even if this hypothesis is not confirmed by data extracted from our population, post-transplant diastolic function remains difficult to define and is largely unknown. Standard parameters such as E/e’, deceleration time, E/A, were found to be unreliable when applied to transplanted hearts, and pathophysiological studies are needed for a better understanding of this field.

It should not be neglected that all the organs that were transplanted on the recipients in our study have been stored in standard cold storage. This technique was proven to be related to graft failure as showed by Collis et al. [50], and could have a worst effect on MD hearts. Looking at the results of the Heart EXPAND trial for the ex-vivo Heart Organ Care System developed by Transmedics, the only existing clinical trial applied on marginal organs, we can tell that the preservation could impact the outcome [51,52]. This study has been designed to retrospectively analyze the data collected, therefore it was not possible to include hearts stored with the ex-vivo System, but the results can strengthen and promote the use of MD together with the OCS Heart. Data on longer follow-up times are needed.

## 5. Limitations

This was a pilot, single-center retrospective study, and therefore it presents some limitations that have to be accounted for. The small sample size of the study does not allow the generalization of the results to a larger population, but it rather represents a starting point for future investigations. Moreover, stress echocardiography performed on potential heart donors might face some technical difficulties due to inadequate acoustic window, which might in turn limit its applicability. The use of contrast medium could be evaluated to optimize acoustic window definition and more sophisticated techniques such as speckle tracking echocardiograpy and myocardial work could improve the morpho-functional analysis of the ventricles. It is also worth considering that all the MD organs of our study were transplanted after static cold storage perfusion. Considering that a great number of MDs are excluded due to ischemic time > 240 min, in addition to the need of enlarging the population with a multi-center involvement, the storage through ex-vivo normothermic perfusion in accurately selected MDs should be tested and compared to standard cold storage.

## 6. Conclusions

Even if our analysis is considered as a pilot study in this field, we place attention on a neglected and often unacknowledged area of discussion in HTX that involves many patients waiting for heart transplantation. The shortage of organs leaves many advanced HF patients without a therapeutic option. The use of organs selected in the “grey area” of heart donors that are excluded from standard criteria because of risk factors that could affect coronary disease or myocardial function could deeply improve survival of waiting list patients. Moreover, the survival of subjects with Left Ventricular Assist Devices (LVADs) remains lower with higher adverse effects and lower quality of life compared to Htx. Coronary angiography is an effective exam when selecting a “high risk” donor but is not applicable on every donor older than 55 or with CV risk factors. We showed how the evaluation of subclincal CAD or cardiomyopathy of marginal hearts with pharmacological echo stress does not affect recipients’ survival compared to controls. The use of a non-invasive and costless exam could therefore be useful to contrast the need of hearts, enlarging the donor list without affecting recipients’ mortality. More studies are needed to expand the use of echo stress in marginal heart selection.

## 7. Clinical Perspectives

The lack of hearts suitable for donations in this era of heart failure therapies amelioration and the elongation of heart transplant waiting lists has become a major issue. The use of marginal hearts has already been proposed but the selection needs to be scrupulous and accurate. The use of non-invasive and reliable techniques such as dipyridamole stress imaging should be considered to uncover acceptable organs and reduce the number of organs rejected. The creation of a network of cardiologic centers is necessary for the training of experts and correct application of the protocol. Moreover, a standard definition of marginal hearts is needed and, consequently, larger populations have to be analyzed to define recipients’ outcomes and demonstrate the accuracy of marginal heart selection.

## Figures and Tables

**Table 1:** Characteristics of Acceptable Heart Donors and Marginal Heart Donors.

Acceptable Heart Donor	
Age	<55 years old
Heart morphology and function	EF 55–65% Normal segmental kinesia No valvular or congenital defects Posterior wall or Interventricular septum < 12 mm
Weight	Maximum 20% donor–receiver weight mismatch
Size	In case of female donor heart for a man: female > 10% larger in height and weight
**Marginal heart donor**	
Age	>55 years old
CV risk factors	High risk (diabetes, arterial hypertension, dyslipidemia, smoking history)
Heart morphology and function	Left ventricular hypertrophy Valvular and/or congenital defects Pre-mature coronary artery disease
Serology	HBV/HCV positive
Substances	Substance intoxication (e.g., CO, cyclic antidepressants) Drug abuse (e.g., cocaine or amphetamine)
Ischemic time	>3–4 h
Size	Elevated donor–receiver size discrepancy (>20%)

*CO: carbonic oxide; CV: cardiovascular; EF: ejection fraction; HBV: hepatitis B virus; HCV: hepatitis C virus.*

**Table 2:** Main differences in baseline characteristics and outcome among recipients of marginal heart donations and acceptable heart donations.

	Total	Marginal	Acceptable
Population	22	11	11
**Baseline characteristics**			
Age	54 ± 10	55 ± 10	54 ± 9
Age of donors	41 ± 23	45 ± 29	36 ± 12
Sex	M 14 (63.6%)	M 7 (63.6%)	M 7 (63.6%)
Sex of donors	M 12 (54.5%)	M 5 (45.4%)	M 7 (63.6%)
NYHA III/IV	3 (13.6%)	0	3 (27.3%)
Arterial hypertension	10 (58.8%)	4 (36.4%)	6 (54.5%)
Diabetes	4 (18.2%)	1 (9.1%)	3 (27.3%)
Dyslipidemia	6 (27.3%)	1 (9.1%)	5 (45.4%)
CKD	11 (50%)	4 (36.4%)	7 (63.6%)
**Follow-up**			
Rejection	5 (22.7%)	1 (9.1%)	4 (36.4%)
Anti-HLA Ab	7 (31.8%)	2 (18.2%)	5 (45.4%)
CAV	7 (31.8%)	1 (9.1%)	6 (54.5%)
Death	4 (18.2%)	1 (9.1%)	3 (27.3%)

*CAV: Cardiac allograft vasculopathy; CKD: chronic kidney disease; anti-HLA Ab: anti-human leukocyte antigen antibodies; M: males; NYHA: New York Heart Association*.

## Abbreviations

CAD: coronary artery disease; CAV: Cardiac allograft vasculopathy; EAE: European Association of Echocardiography; HTx: heart transplant; ICU: intensive care unit; LVADs: left ventricular assist devices; MCS: mechanical circulatory support; WMSI: wall motion score index.
